# Supplementary material for: Time‑resolved multi-omic analysis of paclitaxel exposure in human iPSC‑derived sensory neurons unveils mechanisms of chemotherapy‑induced peripheral neuropathy
Source: Cell Death Dis. 2026 Feb 10;17(1):211. doi: 10.1038/s41419-026-08445-2 (PMC12921266; doi:10.1038/s41419-026-08445-2)
Supplement: Supplementary file 15 — Dataset 1. [file 41419_2026_8445_MOESM15_ESM.zip › CDD_Open_Data/00_Open_Data_Files_Description.docx]

**Open Data Files (CDDIS-25-4526)**

**Time‑resolved multi-omic analysis of paclitaxel exposure in human iPSC‑derived sensory neurons unveils mechanisms of chemotherapy‑induced peripheral neuropathy**

We structured the data files in the order they appear in the Figures and Result section of the manuscript as far as possible.

*01_Microscopic_Files is a folder that contains* the raw file of Fig. 1A.

*02_Cell_Viability_Data* provides the GraphPadPrism File on MTT live viability assay data of Fig. 1B, and Suppl. Fig. 2A. The files 02a_263a_PTX_calculated - 02e_005a_PTX_calculated include the raw data which were incorporated in the GraphPad file. 02f_Averaged_Viabilities includes the averaged viabilities which are given in the Results section.

*03_R_Script_CDD.R* includes all the R-Codes to calculate Fig. 1C, 1E, Fig. 2, Fig. 3, Fig. 4, Fig. 5 and Supplemental Fig. 2, 3, 5. All the Excel-Files containing the respective RNA seq data which are referenced in the R-File are given in 04_RNA_Seq. We created folders in the code with names matching the figures to make its assessment as intuitive as possible (e.g., “#CDD - RNA-Seq Volcano Plots of BIHi264-A, 263-A, 272-A, 273-A, 005-A”, “#CDD - Venn-Diagram and intersect tables of all Cell Lines BIHi263-A, 264-A, 272-A, 273-A, 005-A” etc.).

*04_RNA_Seq* is a folder that harbors the RNA seq excel files, including the differential expression analyses of pooled RNA seq data of all cell lines incubated with paclitaxel vs. DMSO given in Fig. 1C, Fig. 2 (“00_202501_All_lines_contrasts_full.PTX_vs_DMSO_ID0”) and differential expression analyses of all sequential RNA seq data assessed in the course of time from 2h of incubation with paclitaxel vs. DMSO (“01_contrasts_full.2h_PTX_vs_DMSO_ID1”) until 5d after removal of the drugs (“07_contrasts_full.5d_PTX_vs_DMSO_ID6”). RNA seq data of BIHi264-A at 48h is named “05_contrasts_full.48h_PTX_vs_DMSO_ID5”. “All_Cell_Lines_DESeq2.all.rld.blind” contains the RLD of all cell lines. “00_tpms_all_time_points” includes the TPM of all analyzed time points of BIHi264-A from 02h until 5d after removal of the drug. These data were used in Suppl. Fig. 6. “Genes_of_interest_one_list” merges data from “00_202501_All_lines_contrasts_full.PTX_vs_DMSO_ID0” together with the number of overlaps, i.e. significant differential expression of genes between the cell lines. “Gene_expression_over_time” includes the data from Suppl. Fig. 6. The whole pipeline report is to be found in “DE_all_and_otherCL_20241209.tar” (for all cell lines, PTX vs. DMSO 48h). Panel plots, evidence plots and so on from the sequential RNA seq data are packed in “Timeline_panel_plots_etc_2023-01-23_1609.zip”. “Geo_List_RNA_Seq_Data” states all RNA seq raw file names as they were uploaded to the Gene Expression Omnibus (GEO), and “Group_comparison_sheet” the group comparison list for bioinformatical analyses. All raw counts are available on GEO (GEO accession number: GSE312881). “CDDIS-25-4526_MS_GEO.xlsx” contains the Metadata Spreadsheet we uploaded to GEO, stating the exact pooling of samples. All R scripts used for RNA seq analyses are available on <https://github.com/bihealth/seasnap-pipeline>.

*05_Protein* is a folder including the proteomic data with which Fig. 4 was constructed; together with the intensity-based absolute quantification (IBAQs) and the GraphPad File with which Fig. 4A and 4B were created. It also includes additional pathway analyses. R-Script templates for the Disco-Plot in Fig. 4C and D are to be found in *03_R_Script_CDD.R.* The mass spectrometry proteomics data have been deposited to the ProteomeXchange Consortium via the PRIDE partner repository (112) with the dataset identifier PXD070094.

*06_Lipidomics* includes the lipidomic data and GraphPad file with which Fig. 5 was calculated. The respective R file with which the Volcano Plot in Fig. 5C was created is to be found in *03_R_Script_CDD.R.*

*07_Electrophysiology* holds the data on patch clamp experiments, MEA experiments, Ca^2+^ imaging experiments and images of iPSC-DSN on MEA plates. Remark: The calcium imaging experiments for characterization of the cell line BIHi264-A, “264a_Ca_imaging_pooled_from_2021-07-07_2021-07-08_Lois_Christian”, were jointly conducted by Lois Hew and Christian Schinke and are found in the Suppl. Fig. 1D, E of this manuscript and in the Suppl. Fig. 1 of Hew et al., *c-Jun Inhibition Mitigates Chemotherapy-induced Neurotoxicity in iPSC-derived Sensory Neurons* Cell Death Discovery 2025.
